# Propofol Infusion Syndrome: A Retrospective Analysis at a Level 1 Trauma Center

**DOI:** 10.1155/2014/346968

**Published:** 2014-12-17

**Authors:** James H. Diaz, Amit Prabhakar, Richard D. Urman, Alan David Kaye

**Affiliations:** ^1^Critical Care Medicine, Department of Anesthesiology, Louisiana State University Health Science Center, New Orleans, LA 70112, USA; ^2^Environmental and Occupational Health Sciences, School of Public Health, Louisiana State University Health Science Center, New Orleans, LA 70112, USA; ^3^Department of Anesthesiology, Louisiana State University Health Science Center, New Orleans, LA 70112, USA; ^4^Department of Anesthesiology, Perioperative and Pain Medicine, Harvard Medical School, Brigham and Women's Hospital, Boston, MA 02115, USA; ^5^Department of Pharmacology, Louisiana State University Health Science Center, New Orleans, LA 70112, USA

## Abstract

*Objectives.* The propofol infusion syndrome (PRIS), a rare, often fatal, condition of unknown etiology, is defined by development of lipemic serum, metabolic acidosis, rhabdomyolysis, hepatomegaly, cardiac arrhythmias, and acute renal failure.* Methods.* To identify risk factors for and biomarkers of PRIS, a retrospective chart review of all possible PRIS cases during a 1-year period was conducted at a level 1 trauma hospital in ICU patients over 18 years of age receiving continuous propofol infusions for ≥3 days. Additional study inclusion criteria included vasopressor support and monitoring of serum triglycerides and creatinine.* Results.* Seventy-two patients, 61 males (84.7%) and 11 females (15.3%), satisfied study inclusion criteria; and of these, 3 males met the study definition for PRIS, with 1 case fatality. PRIS incidence was 4.1% with a case-fatality rate of 33%. The mean duration of propofol infusion was 6.96 days. A positive linear correlation was observed between increasing triglyceride levels and infusion duration, but no correlation was observed between increasing creatinine levels and infusion duration.* Conclusions.* Risk factors for PRIS were confirmed as high dose infusions over prolonged periods. Increasing triglyceride levels may serve as reliable biomarkers of impending PRIS, if confirmed in future investigations with larger sample sizes.

## 1. Introduction

Propofol is a popular sedative hypnotic commonly used for induction of general anesthesia and sedation in the intensive care unit (ICU). Propofol is a structurally distinct alkylphenol derivative anesthetic agent. It is preferred over other available agents for its rapid onset of action, rapid emergence from sedation, and reduced likelihood of nausea and vomiting. Propofol also provides several key physiological benefits that make its use in the ICU favorable including reduced cerebral metabolic demand, anticonvulsive properties, and neuroprotective effects [1].

Despite these advantages, propofol may rarely cause a potentially fatal condition known as propofol infusion syndrome (PRIS). PRIS was first described in children in the 1990s with several adult case reports following shortly afterwards [2–5]. The term “propofol infusion syndrome” was coined in 1998 when Bray summarized thirteen more propofol-related deaths in children [6]. These children all exhibited a similar constellation of symptoms including metabolic acidosis, lipemic serum, and refractory bradycardia progressing to asystole [7, 8]. In 1996, Merinella was the first author to suggest that a propofol reaction should be included in the differential diagnosis of metabolic acidosis developing in adult patients during long-term sedation with propofol [9]. Subsequently, in 1998, the first case of PRIS in an adult was reported. In this case, nearly all of the earlier presenting signs of PRIS in pediatric patients were described including hypoxia, metabolic acidosis, rhabdomyolysis, renal failure, and cardiac dysfunction [8].

Despite over twenty years of intensive research, the complete pathophysiological mechanisms responsible for PRIS have not been identified. Potential risk factors for PRIS were first identified in five 1992 case reports in relatively healthy children with acute epiglottitis or tracheobronchitis who died after being sedated with propofol in the ICU [2, 3]. In these cases, the pediatric patients who were treated with high dose infusions of propofol over lengthy periods of time developed metabolic acidosis, lipemic serum, and refractory bradycardia progressing to asystole [4–6].

The objectives of this retrospective descriptive study were to better define the incidence and demographics of PRIS in a level 1 trauma center and to identify the most significant risk factors for developing this condition and to further investigate a correlation between increasing triglyceride levels and duration of propofol infusion.

## 2. Patients and Methods

Since this investigation was an anonymous, retrospective chart review based on pharmacy dispensing records, institutional review board approval was not indicated. The present study was a retrospective chart review investigation looking at patients on long duration propofol infusions in the ICU of LSU Interim Hospital in New Orleans. In order to test the null hypothesis of no differences in the demographic characteristics of patients receiving propofol infusions in the ICU for three or more days and no differences in the increases in serum triglyceride and creatinine levels over the duration of propofol infusions, a retrospective anonymous pharmacy database was analyzed for all ICU cases of propofol infusions for three or more days over the 1-year study period, January 1–December 31, 2011. There were 72 patient cases meeting the study inclusion criteria. Other inclusion criteria included age of 18 years or older and nonpregnant status for females. Of the 72 patient cases analyzed, only 3 cases met the study case definition of PRIS, which included evidence of rhabdomyolysis, metabolic acidosis, and cardiovascular depression requiring vasopressor medication. All categorical demographic variables including gender, ethnicity, comorbidities, medications, and vasopressor infusion were compared for any statistically significant differences in their proportions by *z*-tests with statistically significant differences defined by *P* values less than 0.05. Correlation analysis of the increases in serum triglyceride and creatinine levels over propofol infusion durations was conducted by comparing the 2 linear regressions and computing their *r*-values, or Pearson product-moment coefficients, with *r*-values closer to 1.0 reflecting greater correlation over time. The *r*-values that were greater than 0.75 were considered clinically significant. Lastly, an overall *F*-test for the coincidence of the correlation of the increases in serum triglyceride and creatinine levels of propofol infusion durations over time was conducted with statistically significant differences defined by *P* values less than 0.05. All statistical calculations were conducted using the Internet-based, public-domain, R-statistical package available at http://www.r-project.org/.

## 3. Results

A total of 72 patients, 61 males (84.7%) and 11 females (15.3%), met the inclusion criteria for this study. Of these, 3 males met the study definition for PRIS, with 1 case fatality. Although there were no significant differences in the ethnicities between Caucasian and African-Americans and others studied and in the comorbidities and concomitant medications in the cases studied, there were significantly more males (*P* < 0.0001) and more patients receiving analgesics plus sedatives among the cases studied (Table 1). The trauma center PRIS incidence was 4.1% with a case fatality rate of 33%. The mean duration of propofol infusion in all cases was 6.96 days. Since this was a retrospective review of a cohort of cases sedated with prolonged infusions of propofol and not a prospective observational study of similar cases, the 3 cases meeting the study's case definition of PRIS (rhabdomyolysis, metabolic acidosis, and cardiovascular depression) would be defined in a traditional manner as a period prevalence of 4.1% and only a reflection of the true incidence of PRIS in a prospective investigation over a one-year period of observation.

**Figure 1:**
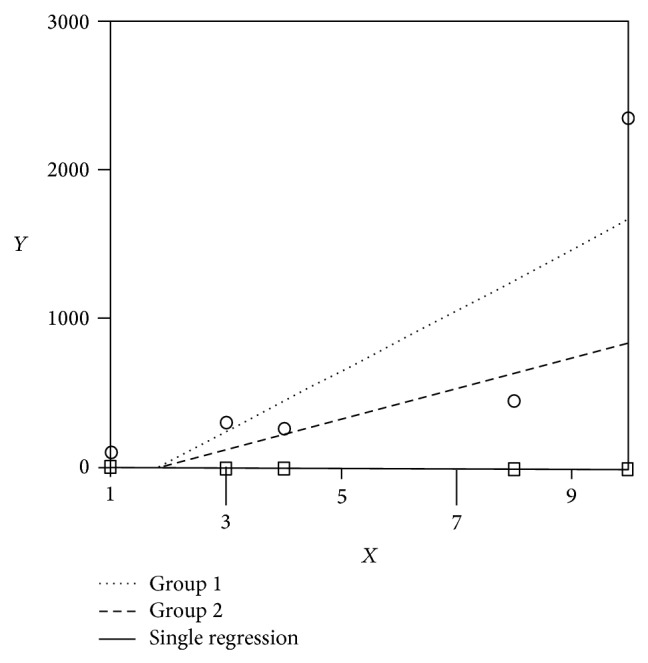
A comparison of the linear regression lines of the increases in serum triglyceride levels over duration of propofol infusion (…) versus increases in serum creatinine levels over duration of propofol infusion (− − −). Note: the creatinine levels (− − −) track the *x*-axis at 0-1 and are reflected in the single regression line. Although there is no difference in the line slopes (*P* = 0.56) or in the *y*-intercepts (*P* = 0.515) of the regression lines, there is a statistically significant difference in the *F*-test of coincidence between the lines indicating significantly greater correlation of increasing triglyceride levels with duration of propofol infusion (*F* = 5.773, *P* = 0.04).

At *r* = 0.807, there was a 100% greater correlational relationship between increases in serum triglycerides over time and the duration of propofol infusion than between increases in serum creatinine over time and the duration of propofol infusion (Table 2). The *F*-test for coincidence demonstrated a statistically significant greater direct relationship between increases in serum triglycerides over time and the duration of propofol infusion than between increases in serum creatinine over time and the duration of propofol infusion (Table 3, Figure 1). Figure 1 displays the visual comparison of the linear regression lines of increases in serum triglycerides over time and the duration of propofol infusion and the increases in serum creatinine over time and the duration of propofol infusion. Of note, the creatinine levels tracked the *x*-axis (0-1), remained within near-normal range (0.5–1.5 mg/dL), and were reflected in the single, combined single regression line (Figure 1). Although there was no difference in the slopes or the *y*-intercepts of the regression lines compared, there was a statistically significant difference in the *F*-test of coincidence between the lines indicating significantly greater correlation of increasing triglyceride levels with duration of propofol infusion (*F* = 5.773, *P* = 0.04) (Figure 1).

## 4. Discussion

The results of the present study demonstrated a 4.1% incidence of PRIS and an overall case fatality rate of 33%. The mean propofol infusion duration was 6.96 days. In the present study, a positive linear correlation was noted between increasing triglyceride levels and duration of infusion, although no significant correlation was observed between increasing creatinine levels and duration of infusion.

In the present study, our trauma center identified a PRIS incidence of 4.1% with a case fatality rate of 33%. However, the true incidence of PRIS is unknown. Roberts described 1,017 critically ill patients receiving propofol infusions for longer than 24 hours in 2009 and found the incidence of PRIS to be 1.1% [9]. Later, the Food and Drug Administration's MEDWATCH system analyzed 1,139 suspected cases of PRIS and estimated an incidence of approximately 30% [10]. In addition to the confusion regarding the true incidence of PRIS, there remains no consensus on the management of PRIS other than early recognition and discontinuation of propofol.

The clinical manifestations that have come to define PRIS are wide ranging and include the development of metabolic acidosis, rhabdomyolysis (skeletal > cardiac), cardiac arrhythmias (including right bundle branch block, Brugada-like syndrome, atrial fibrillation, supraventricular tachycardia, ventricular tachycardia, ventricular fibrillation, and electromechanical dissociation), acute renal failure, lipemic serum, hepatomegaly, and fatal cardiac arrest [6]. Electrocardiographic changes such as coved ST elevations in the right precordial leads may be the first signs of impending cardiovascular collapse in patients receiving propofol infusions [11]. The onset of PRIS may be related to inhibition of intracellular energy production by mitochondria by one or both of the following two mechanisms: (1) inhibition of transportation of long-chain fatty acids into cells during the nutritionally deficient states of critical illnesses and/or (2) inhibitory effects on the intracellular mitochondrial respiratory chain [12].

The metabolic derangements in PRIS appear to be triggered by a combination of (1) metabolic stress and high energy demand during critical illness in susceptible patients; (2) low carbohydrate supplies, especially in children; and (3) a high availability of fats, as seen in propofol's emulsion of soybean oil and egg whites [13, 14]. However, anything that inhibits effective cellular aerobic respiration may result in lactic acidosis that if left untreated may result in rhabdomyolysis, hyperkalemia, and, ultimately, acute renal failure. Other risk factors for PRIS include respiratory infection, severe head injury, propofol sedation for more than 48 hours at doses greater than 4 mg/kg/hr, and increased catecholamine and glucocorticoid serum levels [11]. A retrospective cohort study from an adult neurosurgical ICU showed the importance of this dose-dependent relationship [15]. Cremer et al. found that no patients showed signs of PRIS at <5 mg/kg/h. However at 5-6 mg/kg/h there was a 17% incidence and a 31% incidence at >6 mg/kg/h [16]. A meta-analysis by Fong et al. found that death from PRIS was more likely if a patient was younger than 18, received a vasopressor, or developed any of the following symptoms: cardiac arrhythmias, rhabdomyolysis, impairment in renal function, metabolic acidosis, or dyslipidemia [10].

Propofol sedation paired with catecholamine use may have both direct and indirect consequences on cardiovascular and skeletal muscle integrity [17]. Researchers used an ovine model to study the physiologic and pharmacologic perturbations with concomitant propofol and catecholamine infusions [18]. While catecholamines significantly increased cardiac output, propofol blood concentrations linearly decreased from baseline [17, 18]. In some instances, propofol blood concentrations were reduced so greatly that reversal of anesthesia was observed. Researchers attributed the decreased blood levels to increased first pass metabolism and clearance secondary to increased cardiac output [17, 18]. This may explain why patients on vasopressors require higher doses of propofol to maintain sedation and subsequently may have a higher correlation to PRIS development [19]. Higher doses of propofol may be associated with both myocardial and skeletal muscle damage subsequently resulting in eventual cardiovascular collapse.

A differential diagnosis of PRIS is presented in Table 4. Congenital cardiac conduction disorders, such as Brugada Syndrome, an autosomal dominant disorder in right ventricular conduction, can result in sudden death. Genetic polymorphisms that affect lipid metabolism, such as medium-chain acyl-coenzyme A dehydrogenase deficiency, may also be risk factors for PRIS and should be included in the differential diagnosis [20, 21]. Medications can also be a cause of laboratory derangements resembling PRIS. The medications that should be considered when ruling out PRIS either have the capability to cause muscle injury or have been shown to cause renal damage. Finally, miscellaneous conditions that need to be considered in the differential diagnosis of PRIS include the use of typical antipsychotics in bipolar patients and the presence of ongoing seizures in an epileptic patient, both of which can cause rhabdomyolysis [21].

There are several possible preventive measures clinicians should consider while administering continuous propofol infusions. These measures are based on the proposed pathophysiologic principles of the syndrome. Critically injured patients often have an inadequate supply of carbohydrates, resulting in increased fat mobilization and usage [22]. This increase in circulating fatty acid can predispose patients to PRIS [23]. Maintaining adequate carbohydrate intake in critically ill patients may prevent the switch to fat metabolism and thus prevent the onset of PRIS [24, 25]. Pediatric patients have much smaller carbohydrate stores relative to adults. This concept may explain the lower incidence of PRIS in adults. Researchers have proposed that a carbohydrate intake of 6–8 mg/kg/min can suppress fat metabolism and possibly prevent PRIS [24]. The amount of lipid in the propofol formulation may also contribute to PRIS onset [22]. The use of a more concentrated propofol solution may help to reduce this lipid burden [26].

The strengths of this retrospective study are the use of a pharmaceutical database developed to track the use and costs of medications and to identify any potential drug-drug interactions. In addition, we used a preexisting clinical case definition for PRIS and well-defined inclusion and exclusion criteria. We also had an equally distributed Caucasian and non-Caucasian population, being able to eliminate it as an effect modifier. There are some limitations to the study. As a retrospective review, we did not have a control group, such as a cohort of dexmedetomidine-sedated patients. We have a relatively small sample size of 72 patients, and ideally we should have measured the creatine kinase levels over the duration of the propofol infusions to pick up early biomarker evidence of rhabdomyolysis. We had limited data on the patient's diagnosis and were unable to stratify our study cohort by the two most common effect modifiers: age and gender.

In conclusion, a presumptive diagnosis of PRIS will include clinical and laboratory confirmation of a constellation of rhabdomyolysis, hyperkalemia, hyperlipidemia, and ARF in adults receiving high dose propofol infusions (>4 mg/kg/h) for prolonged (>48 h) periods. Although our retrospective chart review had a case fatality rate for PRIS of 33% consistent with other investigations, the study's incidence rate of 4.1% was four times higher than the 1.1% incidence rate in Roberts 2009 prospective study. This considerable difference in healthcare facility incidence may be accounted for by several key variables including more trauma patients with soft tissue injuries, more trauma patients with head injuries requiring prolonged sedation for mechanical ventilation, and the greater use of propofol over other sedative hypnotics for sedation in the ICU.

PRIS remains a complex and multifaceted clinical syndrome, and the overwhelming majority of patients diagnosed with PRIS have significant preexisting and overlapping comorbidities. Patients with undiagnosed defects in long-chain fatty acid transport and mitochondrial energy production during critical illnesses may be at significantly increased risks of PRIS. Propofol should not be used for sedation for more than 3 days if possible. During propofol infusions, clinicians should monitor arterial blood gases, serum triglycerides, creatine kinase, all electrolytes (particularly potassium), serum lactate levels, liver function tests, blood urea nitrogen, and creatinine.

Over twenty years of intensive research has failed to demonstrate the complete pathophysiological mechanisms responsible for propofol infusion syndrome. The diagnosis of PRIS is usually by exclusion because patients often exhibit other potentially fatal comorbidities including metabolic acidosis, acute renal failure, and rhabdomyolysis. Anesthesiologist and intensivists should remain vigilant for the various clinical manifestations of PRIS as prompt identification and cessation of propofol use is essential to maximize patient survival. Future large prospective, randomized controlled trials comparing outcomes of several sedation protocols in ICU patients will be needed to determine the true incidence of PRIS, to identify genetically susceptible patients, and to develop clinical guidelines for propofol sedation without increasing risks of PRIS.

## Figures and Tables

**Table 1:** Results: patient demographics (*N* = 72).

Gender (*n*)	Ethnicity (*n*)	Comorbidities (*n*)	Medications (*n*)	Vasopressors (*n*)
Male (61) Female (11)	Caucasian (36) African-American (29) Hispanic (3) Asian (2) Others (2)	Diabetes (9) Hyperlipidemia (13)	Analgesics + sedatives (71) Total parenteral nutrition (12) Antipsychotics (5)	Dopamine (10) Norepinephrine (5)
*z* = 4.608	*z* = 0.236	*z* = 0.240	*z* = 6.674	*z* = 1.566
*P* < 0.0001^*^	*P* = 0.814	*P* = 0.818	*P* < 0.0001^*^	*P* = 0.177

^*^Statistically significant differences in demographic proportions compared, *P* < 0.05.

**Table 2:** Results: correlation of increases in serum triglyceride and creatinine levels over propofol infusion durations.

	Linear regression of serum triglyceride levels	Linear regression of serum creatinine levels	Combined single linear regression line
*n*	5	5	10
Slope	205	0.006	103
*r* (Pearson product-moment correlation coefficient)	0.807^*^	0.419	0.492

^*^At *r* = 0.807, there is a 100% greater relationship between increases in serum triglycerides over time and the duration of propofol infusion than between increases in serum creatinine over time and the duration of propofol infusion.

**Table 3:** Results: overall test for the coincidence of the correlation of increases in serum triglyceride and creatinine levels over propofol infusion durations.

	Linear regression of serum triglyceride levels	Linear regression of serum creatinine levels	Combined single linear regression line
*n*	5	5	10
Slope	205	0.006	103

*F*-test value	*F* = 5.773		
*P* value	*P* = 0.04^*^		

^*^Statistically significant, *P* < 0.05. At *P* = 0.04, there is a significantly greater relationship between increases in serum triglyceride over the duration of propofol infusion than the relationship between increases in serum creatinine over the duration of propofol infusion, even though both measurements demonstrate positive increases in serum levels over time.

**Table 4:** Differential diagnosis of PRIS.

Congenital	Metabolic	Medications	Miscellaneous
Brugada Syndrome MCADD^*^ Hereditary monogenic disorders	Hypoperfusion Hypoxia Sepsis Diabetic ketoacidosis	HMG-CoA reductase inhibitors Corticosteroids Renal toxic antibiotics ACE inhibitors/angiotensin receptor blockers Renal toxic chemotherapeutic agents Cimetidine Protease inhibitors	Direct muscle injury Hypoxia from traumatic lung injury Seizures Immobilization Myoclonus Neuroleptic malignant syndrome Contrast induced acute kidney injury

^*^MCADD (medium-chain acyl-coenzyme A dehydrogenase deficiency).
